# Molecular Autopsy for Sudden Death in the Young: Is Data Aggregation the Key?

**DOI:** 10.3389/fcvm.2017.00072

**Published:** 2017-11-09

**Authors:** Manuel Rueda, Jennifer L. Wagner, Tierney C. Phillips, Sarah E. Topol, Evan D. Muse, Jonathan R. Lucas, Glenn N. Wagner, Eric J. Topol, Ali Torkamani

**Affiliations:** ^1^The Scripps Translational Science Institute, Scripps Health, The Scripps Research Institute, La Jolla, CA, United States; ^2^Division of Cardiology, Scripps Clinic, La Jolla, CA, United States; ^3^Medical Examiner Department, San Diego County, San Diego, CA, United States

**Keywords:** molecular autopsy, sudden cardiac death, whole exome sequencing, gene panel, mitochondrial DNA

## Abstract

The Scripps molecular autopsy study seeks to incorporate genetic testing into the postmortem examination of cases of sudden death in the young (<45 years old). Here, we describe the results from the first 2 years of the study, which consisted of whole exome sequencing (WES) of a cohort of 50 cases predominantly from San Diego County. Apart from the individual description of cases, we analyzed the data at the cohort-level, which brought new perspectives on the genetic causes of sudden death. We investigated the advantages and disadvantages of using WES compared to a gene panel for cardiac disease (usually the first genetic test used by medical examiners). In an attempt to connect complex clinical phenotypes with genotypes, we classified samples by their genetic fingerprint. Finally, we studied the benefits of analyzing the mitochondrial DNA genome. In this regard, we found that half of the cases clinically diagnosed as sudden infant death syndrome had an increased ratio of heteroplasmic variants, and that the variants were also present in the mothers. We believe that community-based data aggregation and sharing will eventually lead to an improved classification of variants. Allele frequencies for the all cases can be accessed *via* our genomics browser at https://genomics.scripps.edu/browser.

## Introduction

More than 10,000 individuals under the age of 45 years die suddenly and unexpectedly in the USA each year (1). While ischemic heart disease remains the predominant cause of cardiac arrest in the older population, most sudden deaths in the young (SDY) stem from cardiomyopathies (including channelopathies) (2, 3). SDY remains an elusive entity for the medical community, mostly because death is often the first manifestation of the disease. Postmortem forensic examination reveals a pre-existing structural abnormality on the heart (a.k.a., autopsy positive) in approximately two-thirds of the cases, however, for the remaining one-third of the cases no cause of death is identified by traditional postmortem examination (a.k.a., autopsy negative) (4–7). The lack of an identifiable diagnosis after autopsy leaves family members without an explanation for the cause of death of their relative and potentially at risk themselves despite having no manifestations of disease.

Many of the underlying causes of SDY are hereditary, thus, a postmortem genetic diagnosis [molecular autopsy (MA)] provides a landmark for both the identification of the cause of death and a potential resolution of the uncertainty for risk to living relatives (8–13). In this regard, minimally invasive molecular tests, enabled by recent technological advances in high-throughput DNA sequencing, have reduced the cost of genomic sequencing relative to the gene panel-based tests traditionally available to medical examiners (14–17). Thus, the MA represents an exciting opportunity to fill in or supplement the knowledge gaps from traditional clinical autopsies while potentially providing accurate genetic information that could facilitate prevention—especially in cases of SDY (6, 18–28).

In this light, we initiated the first systematic and prospective, family-based, MA study in 2014, jointly organized by the San Diego County medical examiner’s (ME) office and our academic medical center. In this study, whole exome sequencing (WES) of participants in addition to family members (preferably parents) was assessed for potential heritable causes of sudden death. Our preliminary analysis from 2016 (29) performed in 25 cases lead to a positive diagnosis—identification of a known or expected pathogenic in a gene related to sudden cardiac death (SCD)—in 20% of the cases. We also observed that variant interpretation and classification was complicated by the lack of a robust database of sudden death-associated genetic mutations. Since then, our efforts have been focused on recruiting other ME centers throughout the nation (e.g., Onondaga County, NY, USA; Las Vegas, NV, USA; Grand Rapids, MI, USA) to establish a repository of genetic variants that will allow for large-scale genetic studies and empower disease prevention.

Three years after initiating our program, we had sequenced 50 sudden death cases including autopsy negative (e.g., sudden infant death syndrome) and autopsy-positive [e.g., hypertrophic cardiomyopathy (HCM), ruptured aneurysm, etc.] cases and interpreted the results. Here, we describe major findings from the study, emphasizing data aggregation, with the objective of providing a broad view of the genetic causes of SDY.

## Materials and Methods

### Recruitment, Screening, and Consent

The Scripps Institutional Review Board approved the Scripps Molecular Autopsy Study (IRB-14-6386) in 2014. Informed consent was obtained from all living participants or from next of kin for deceased individuals. Individuals between birth and 45 years of age presenting with sudden unexpected death (autopsy-positive cases: where a previously undiagnosed structural abnormality of the heart or other viscera was reported by the ME) or sudden unexplained death (autopsy-negative cases: where no significant findings related to SDY were reported by the ME) etiology were eligible for enrollment. Deaths from an external cause or in persons with known comorbid chronic conditions are excluded. A full description of the inclusion and exclusion criteria can be found at http://clinicaltrials.gov (trial ID: NCT02168088). The San Diego ME’s Office and our research team screened each case for eligibility. In order to be accepted, the forensic autopsies needed to have external and internal examination of the body, as well as histological (including microscopic examination) and toxicological analysis (if requested). Additional cases from non-San Diego offices were screened upon request.

### Sequence Data Generation, Analysis, and Interpretation

Once the cases were selected and informed consent was obtained, biological samples were to our lab at the Scripps Translational Science Institute for sequencing of the proband and biological family members. WES was performed on blood samples from the proband, whereas saliva samples were used for parents (when available) in cases where the proband was <18 years old. Full details of exome sequencing, analysis, and interpretation methodology have been described elsewhere (30). Briefly, exome sequence data generation was performed *via* standard Illumina HiSeq2500 using an Agilent SureSelect target enrichment exome capture that yielded a median coverage of 48× per sample (interquartile range: 43–52, min value: 43, max value: 52) Data analysis was performed according to the Genome Analysis Toolkit variant calling (GATK) best practices (31). After GATK stage, all samples were analyzed for internal quality control (coverage statistics, gender match, relatedness in trios, etc.) with state-of the-art tools (32, 33). Variants were then filtered, annotated, and categorized with our SG-ADVISER tools (34) in three categories: (a) likely causal (mutation previously reported or expected pathogenic in a SCD-related gene), (b) plausibly causal (mutation of unknown significance in an SCD gene), and (c) speculative (mutation previously reported in other disorders) cause of death. A MA research report was considered *positive* when at least one DNA variant was identified as a likely cause of sudden death. A report was considered *negative* when no DNA variants that definitively or likely explain the cause of sudden death were identified. Sanger sequencing was utilized to confirm candidate causal variants (35).

## Results

### Cohort Statistics

From August 2014 to March 2017, we compiled 50 cases, with ages ranging from 2 months to 44 years (Table 1; Table S1 in Supplementary Material), of which 19 were females and 31 were males (1:2 ratio). One of the cases (MA00024) consisted of two male sibling probands (01P and 02P), but, from the two, only the autopsy negative (02P) was included in the global analyses. When the proband was <18 years old we also performed WES on the biological parents (if available) to empower trio-based variant calling analysis that enabled identification of *de novo* variants. In the two cases where family segregation of potentially informative phenotypes such as palpitations or syncope existed (MA00024 and MA02002), we also sequenced other relatives. Due to the disparity of ages in the cases and the consequential effect of aging on the phenotypes, for analysis purposes we split the cohort in three overlapping groups (Table 1): (i) infants (*n* = 8; ages from 0 to 12 months), (ii) trios (*n* = 17; ages from 0 to 18 years), and (iii) full cohort (*n* = 50). Unless otherwise stated, only variants from the proband are displayed. The San Diego cohort (cases labeled MA0[01]xxx in Table 1) did not show any enrichment for any specific race and is representative of the demographic distribution of the County (31 cases: 52% White, 29% Hispanic, 13% African-American, and 6% Asian). Other states demographic trends were not analyzed due to the small sample size.

**Table 1:** Summary of the demographic characteristics and autopsy findings in the 50 cases of sudden death in the young from our study.

ID	Age	Sex	Race	Forensic autopsy	Forensic autopsy summary	Molecular autopsy	Likely causal variants	Variant phenotype
MA00072	2 months	Male	Biracial	Negative	SIDS (bronchopneumonia following resuscitation for SIDS)	Negative		
MA00002	3 months	Male	White	Negative	SIDS	Negative		
MA00005	3 months	Male	Asian	Negative	SIDS	Negative		
MA00011	3 months	Male	White	Negative	SIDS	Positive	NM_001035.2(RYR2):c.7601T>C (p.Leu2534Pro)	CPVT
MA00019	3 months	Male	Hispanic	Negative	SIDS	Positive	NM_002977.3(SCN9A):c.2971G>T (p.Val991Leu)^a^	Epilepsy
MA00056	8 months	Female	Hispanic	Negative	SIDS	Negative		
MA00017	9 months	Male	African-American	Negative	SIDS (hippocampal abnormality/recent history of possible seizures)	Positive	NM_001293307.2(SCN10A):c.742G>C (p.Asp248His)	BrS
MA00007	10 months	Male	White	Negative	SIDS	Negative		
MA02005	1	Male	White	Negative	SIDS	Positive	NM_005477.2(HCN4):c.2275G>A (p.Val759Ile)	BrS
MA00029	2	Female	Hispanic	Positive	Aneurysm (rupture of left middle cerebral artery aneurysm)	Negative		
MA02009	2	Female	NA	Negative	SIDS (febrile seizure)	Negative		
MA02001	9	Male	NA	Negative	SUXD	Negative		
MA02008	10	Female	NA	Negative	Complication from chronic lymphocytic thyroiditis with T3 thyrotoxicosis	Negative		
MA02004	15	Male	White	Positive	Borderline cardiomegaly/valve dilation	Negative		
MA02007	17	Female	NA	Positive	HCM	Positive	NM_000257.3(MYH7):c.4377G>T p.K1459N	HCM
MA04006	17	Male	White	Negative	–	Negative		
MA00024	17/24	Males	Hispanic	Positive/Negative	HCM/–	Negative/negative		
MA00080	19	Female	African-American	Negative	SUDEP	Negative		
MA00025	21	Male	NA	Negative	SUXD	Negative		
MA02003	21	Male	NA	Negative	–	Negative		
MA00016	22	Male	Hispanic	Positive	HCM	Negative		
MA00081	22	Male	White	Negative	SUXD	Negative		
MA03003	23	Female	Asian	Positive	HCM/increased fatty infiltration of RV/enlarged heart	Negative		
MA04004	24	Male	White	Positive	RV cardiomyopathy/fibrofatty replacement RV/dilated RV	Negative		
MA04005	24	Female	White	Negative	Moderate mitral valve prolapse/fibrosis LV	Negative		
MA00038	25	Female	White	Positive	ARVC/D	Positive	NM_004572.3(PKP2):c.1132C>T (p.Gln378Ter)	ARVC/D
MA00098	25	Male	White	Positive	DCM	Negative		
MA00066	27	Female	African-American	Positive	Aneurysm (ruptured cerebral artery)	Negative		
MA02010	27	Male	African-American	Negative	SUXD/moderate RV dilation	Positive	NM_000371.3(TTR):c.424G>A (p.Val142Ile)	Amyloidosis
MA00018	28	Male	African-American	Positive	HCM	Negative		
MA02002	29	Male	White	Positive	Coronary artery atherosclerosis	Positive	NM_017636.3(TRPM4):c.1575G>A (p.Trp525Ter)	Progressive familial heart block
MA00052	30	Male	Hispanic	Positive	SCD/myocardial ischemia/cardiac arteriolosclerosis and apparent vasospasm	Positive	NM_005751.4(AKAP9):c.11272C>T (p.Arg3758Cys)	LQTS
MA00068	30	Male	White	Positive	DCM	Negative		
MA00003	31	Male	White	Negative		Negative		
MA00047	32	Male	Hispanic	Negative	SUXD	Negative		
MA01001	32	Male	White	Positive	HCM	Positive	NM_002471.3(MYH6):c.3010G>T (p.Ala1004Ser)	HCM
MA00027	33	Male	White	Positive	SUD (arteriosclerotic cardiovascular disease/thrombotic occlusion of left anterior descending artery)	Positive	NM_000361.2(THBD):c.302G>T (p.Arg101Leu)	Thrombophilia
MA00033	34	Female	Hispanic	Positive	Aneurysm (probable ruptured berry aneurysm/acute non-traumatic subarachnoid hemorrhage/HCM/fatty change of liver)	Negative		
MA00054	36	Female	Asian	Positive	SCD/acute coronary artery dissection/eosinophilic coronary arteritis	Negative		
MA03006	36	Male	White	Positive	Coronary artery atherosclerosis	Negative		
MA00055	37	Female	White	Positive	HCM and ischemic brain injury	Negative		
MA04007	37	Female	White	Positive	Healed viral myocarditis/RV cardiomyopathy	Positive	NM_004415.3(DSP):c.268C>T (p.Gln90Ter)	ARVC/D
MA00076	38	Female	White	Negative	–	Negative		
MA00082	39	Female	White	Negative	SUDEP	Negative		
MA00032	40	Male	White	Positive	Hypertensive and atherosclerotic cardiovascular disease/hepatomegaly	Negative		
MA00048	41	Female	White	Positive	Hypertensive cardiovascular disease/acute left basal ganglia hemorrhage (stroke)	Negative		
MA02006	41	Female	White	Positive	HCM/mild myocardial fibrosis	Negative		
MA00046	43	Male	Hispanic	Positive	HCM (mild hypertensive cardiomyopathy and obesity)	Positive	NM_001035.2(RYR2):c.9673G>A (p.Gly3225Ser)	CPVT
MA00001	44	Male	White	Positive	HCM	Positive	NM_017636.3(TRPM4):c.1697C>T (p.Ala566Val)/NM_006514.3(SCN10A):c.3704C>T (p.Ala1235Val)	Progressive familial heart block/BrS
MA02000	44	Female	White	Positive	DCM	Negative		

*Note that Table S1 in Supplementary Material contains an extended version of this table*.

*ARVC/D, arrhythmogenic right ventricular cardiomyopathy/dysplasia; BrS, Brugada syndrome; CPVT, catecholaminergic polymorphic ventricular tachycardia; DCM, dilated cardiomyopathy; HCM, hypertrophic cardiomyopathy; LQTS, long QT syndrome; NA, not available; LV, left ventricle; RV, right ventricle; SCD, sudden cardiac death; SIDS, sudden infant death syndrome; SUDEP, sudden death in epilepsy*.

*^a^Homozygous variant*.

**Figure 1:**
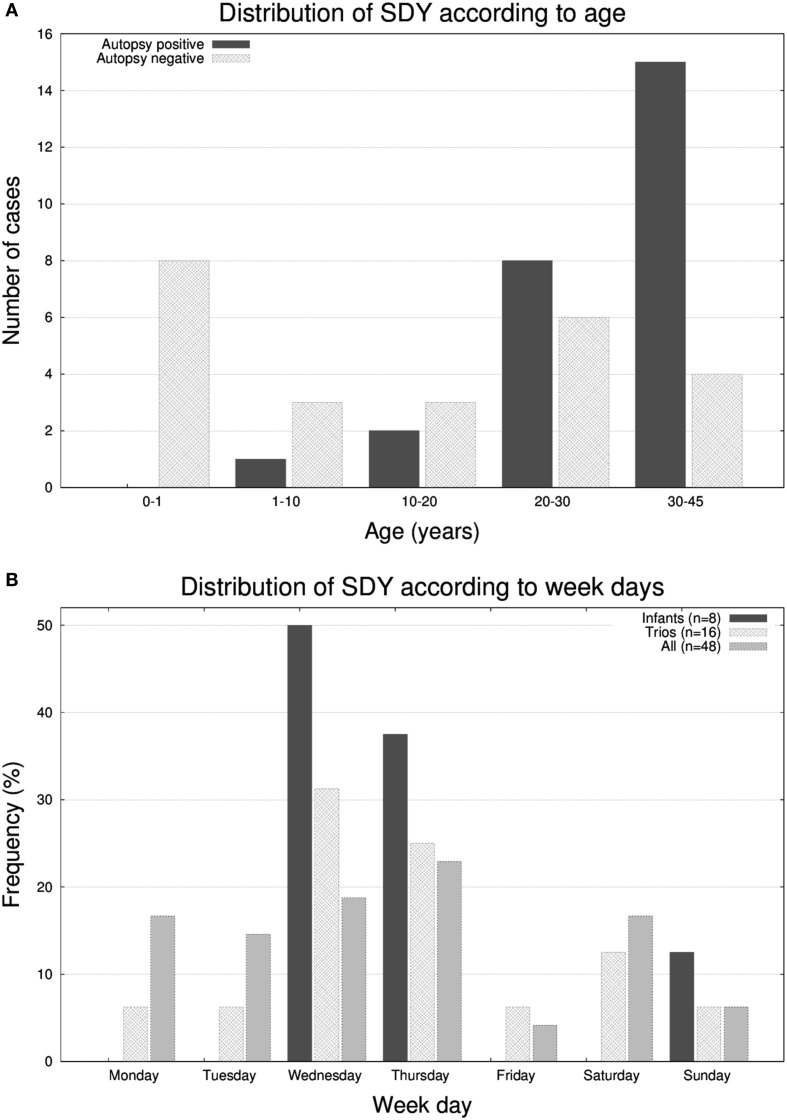

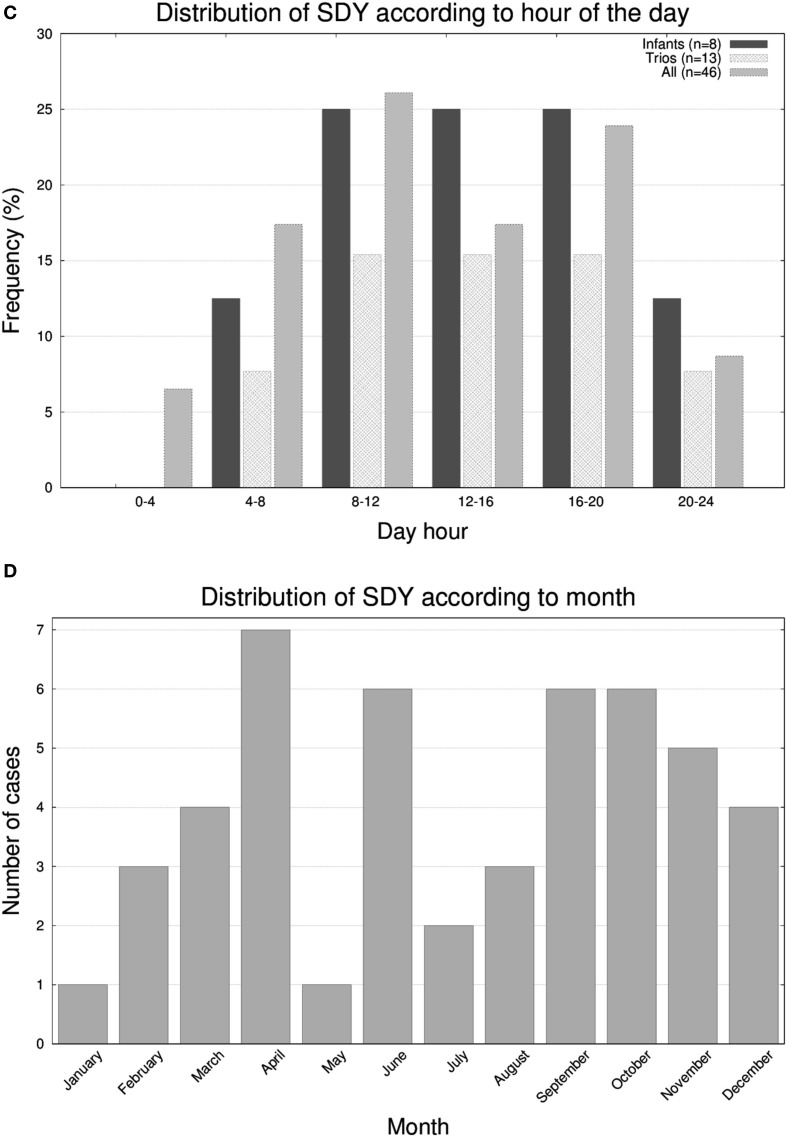
Histograms representing the number of molecular autopsy cases of sudden death in the young (SDY) according to **(A)** 6 group ages, **(B)** day of the week, **(C)** hour of the day, and **(D)** month of the year.

The cohort had a bimodal distribution of ages (Figure 1A). There was a peak for infant cases (eight cases) and then an exponential increase peaking with participants of 30–45 years of age. All forensic autopsies were negative for infants, transitioning to positive with increase in age. For the entire cohort, 52% (26 out of 50) of the forensic autopsies (FA) were positive. The FA+ consisted of 10 cases (38%) of HCM, 3 cases (12%) of aneurism, 3 (12%) cases associated with ischemic events due to coronary artery disease (not expected to be significant enough to cause sudden death), and the remaining 10 cases associated with other causes (Table 1; Table S1 in Supplementary Material). In terms of molecular autopsies, 28% (14 out of 50) cases were positive and 72% came out as negative. In this regard, a common concern is whether molecular autopsies improve the diagnostic yield over forensic ones (26). In Table 2, we compared the diagnostic yield from all four possibilities that appear when combining both types of autopsies (molecular/forensic). According to our data, 19 out of 50 cases (38%) were −/−, whereas 9 (18%) were +/+. Interestingly, the number of cases where MA could not help was around the same for FA+ and FA− cases (17 and 19, respectively, out of 50). Molecular autopsies found a likely causal variant in 9 FA+ cases and 5 FA− cases.

**Table 2:** Combined diagnostic yield for forensic autopsies and molecular ones.

	Forensic autopsy	
Molecular autopsy	+ (*n* = 26)	− (*n* = 24)
+ (*n* = 14)	9	5
− (*n* = 36)	17	19

It has been reported that SCDs follow circadian/diurnal/seasonal variation (1). To evaluate these trends in our cohort, we represented histograms of the frequency of deaths according to the day of the week (Figure 1B), hour (Figure 1C), and month (Figure 1D). Unexpectedly, we found a substantial accumulation of sudden unexplained deaths during the middle of the week in the very young population (seven out of eight infants passed away on Wednesdays or Thursdays; *p*-value <0.05). With respect the hour of the day, most deaths occurred in the 8 a.m. to 8 p.m. 12-h window, and, in terms of seasonality, we observed two peaks, one in the month April and another around early Fall.

### Variant Distribution in Genes: The Case for Exome versus Gene Panel

Two of the most interesting observations that stem from WES of disease cohorts are the qualitative (location in the genome) and quantitative analysis of pathogenic variants. In our case, after applying GATK’s variant calling pipeline we obtained a median value of 98,667 variants per sample (see full statistics in Table S2 in Supplementary Material; aggregate data for all cases can be accessed at https://genomics.scripps.edu/browser). From these, a median of 80,445 variants per sample passed quality controls. The variant calling format (VCF) files generated were subsequently parsed with our additional *in-house* filter to discard common variants [variants with a minor allele frequency (MAF) ≥5% in our internal database]. This filter removed a very high percentage of the variants, ending up with a median of 4,684 variants per sample. The final step involved again filtering [keeping only variants with MAF < 1% in 1,000 genomes (36)], plus annotation and prioritization of variants according to the SG-ADVISER protocol (34) leading to a median of 354.5 variants per sample. After applying all these filters, only ~0.4% of the variants were retained from the original VCFs coming from GATK’s pipeline.

Exome sequencing was performed as a first pass in all 50 cases. However, many other laboratories performing varieties of molecular autopsies use more affordable methods such as gene panels specific for heart disease (23, 25, 26, 37). In that regard, a common topic of debate is whether or not “escalating” to exome helps in deciphering more cases, or if it simply adds unnecessary complexity to an already intricate procedure. To shed some light in this issue, here we analyzed the VCF files resulting from WES at both gene panel and exome level. In terms of a gene panel, we restricted the variant search to an *ad hoc* list of genes (labeled as “panel”) consisting of 233 relevant loci associated with heart disease (see full list of genes in Table S3 in Supplementary Material). For exome, we did a full search without restrictions to particular loci (labeled as “exome”).

**Figure 2:**
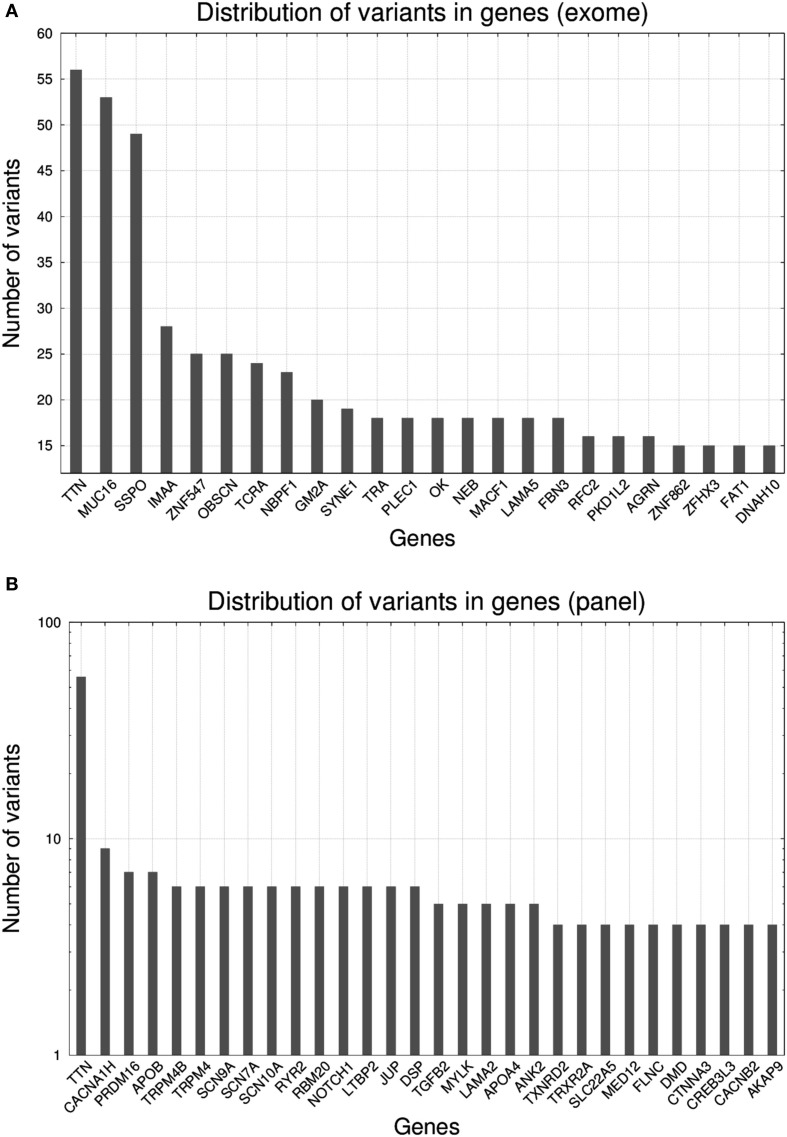

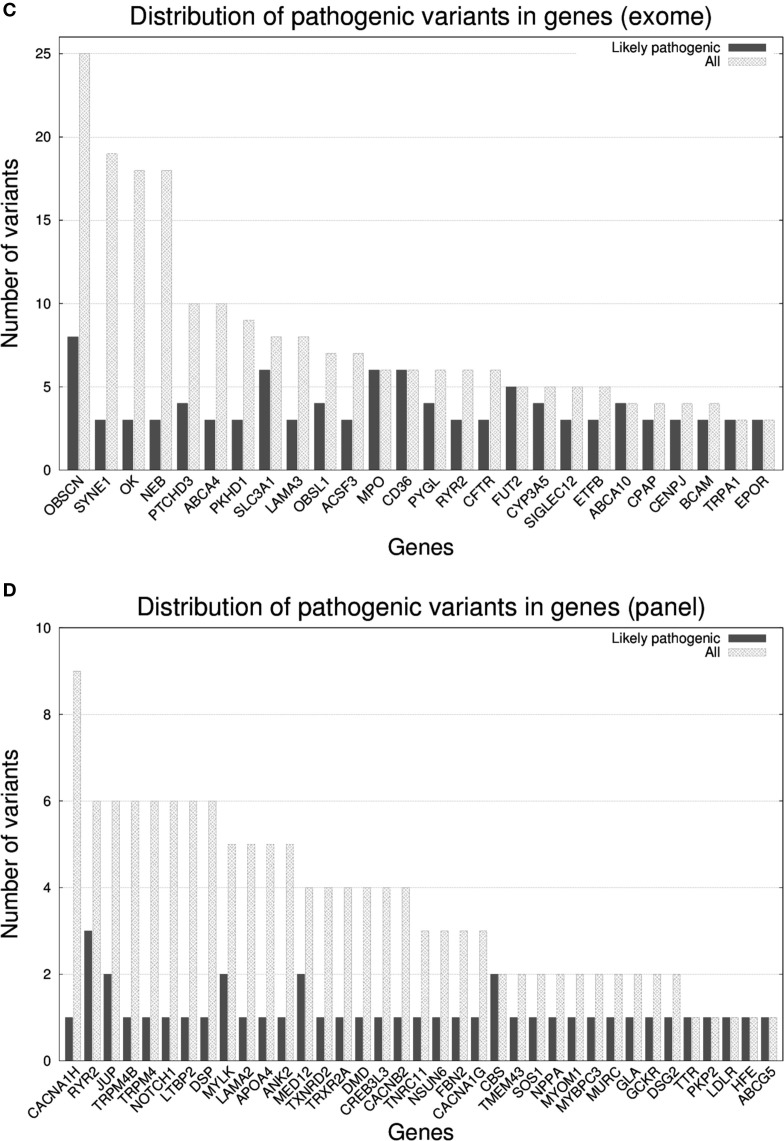
Histograms representing the number of variants per gene* for 50 molecular autopsy cases: **(A)** when taking into account all exome genes**, **(B)** when taking into account a gene panel consisting of 233 genes associated to sudden death (note log_10_ transformation in *y*-axis), **(C)** when taking into account exome genes with ≥3 likely pathogenic variants, **(D)** when taking into account panel genes with ≥1 likely pathogenic variant. *Non-coding RNA loci were excluded from the figures (e.g., *AK093733*). **Fourteen *PCDHG[AB][1-9]* loci (protocadherin gamma subfamily A and B) were excluded yet in total they carried 367 variants.

In Figure 2, we present the abundance of variants per gene according to exome (Figure 2A) and gene panel (Figure 2B). The transition from exome to gene panel creates an important reduction in the number of variants obtained, going from a median of 354 per sample in exome (interquartile range: 325–396, min value: 235, max value: 739, total number of variants for the 50 cases was 19,505) to a median of 7 (interquartile range: 5–10, min value: 1, max value: 18, total number of variants for the 50 cases was 374). For exome, variants in *TTN* were the most abundant (56 variants), followed by *MUC16* (53 variants) and *SSPO* (49 variants). *TTN* gene encodes titin, the largest protein of the human genome [305 kilobases (kb)] a protein involved in many cardiomyopathies (38). *MUC16* (mucin 16) is a 132 kb gene encoding the mucin protein, a glycoprotein associated with cancers, whereas *SSPO* (subcommissural organ spondin) is a 58 kb gene that encodes a protein involved in the modulation of neuronal aggregation. Due to their large size, both *TTN* and *MUC16* are frequently seen in exomes and caution is advised with the interpretation of variants (39). For *TTN*, the median number of variants per sample was 1 (interquartile range: 0–2, min value: 0, max value: 7), for *MUC16* the median number was 1 (interquartile range: 0–1, min value: 0, max value: 13), and for *SSPO* the median number was 0 (interquartile range: 0–1, min value: 0, max value: 14). We found that MA00066, the case with the largest number of variants (739), carried 7 variants in *TTN*, 14 variants in *SSPO*, and 13 variants in *MUC16*. Case MA00066 is a 27-year-old African-American female for whom the cause of death was reported as a ruptured cerebral artery aneurysm. None of the variants in those three genes was annotated to be pathogenic, and actually our MA research report for this case came out as negative for nuclear variants. For the gene panel, we found again that *TTN* was the most abundant, with a 10-fold increase with respect the rest of the genes.

Due to the inherent difficulty of analyzing all exomic variants in a given sample, researches usually resort to methods that prioritize variants according to predicted pathogenicity. According to this, we restricted the search to variants assessed as likely pathogenic according SG-ADVISER (34) clinical categories 1 (variant is previously reported and is a recognized cause of the disorder), and 2 (variant is previously unreported and is of the type that is expected to cause the disorder). SG-ADVISER categories 1–2 overlap with the American College of Medical Genetics scoring guidelines [see discussion in Ref. (34)]. For exomes, the median number of likely pathogenic variants was 10.5 (interquartile range: 8–13, min value: 5, max value: 20) whereas for the gene panel we found that 23 out 50 samples (46%) had zero variants, 18 samples had 1 (36%), 6 samples had 2 (12%), and 3 samples had 3 (6%). According to these numbers, in the context of coding regions, “de-escalating” from exome to gene panel imposes an upper limit in the yield of positive molecular autopsies, as only 54% of cases analyzed with the gene panel carried a likely pathogenic variant. Conversely, when working at exome level, even the individual with the lowest number of likely pathogenic variants carried five of them (yet most variants were in genes *a priori* not associated with sudden death). With respect the effect of the mutations, for exome we identified 382 missense variants, 49 nonsense variants, and 74 frameshift variants, whereas for the gene panel, 32 were missense variants, 5 nonsense variants, and 2 were frameshift variants.

As seen previously (29), many variants that did not fall under the likely pathogenic category, ended being classified as variants of unknown significance (VUS). A high percentage of them were previously unreported in online variant databases such as ClinVar (40); thus, any source of information that facilitates their classification becomes fundamental. In that sense, estimation of the ratio of pathogenic variants per gene versus the total can be used a reference (41–43). In Figure 2C (exome) and Figure 2D (gene panel), we reported this ratio with a histogram and we observed that there existed genes (e.g., *OBSCN, SYNE1, OK*, and *NEB* in exome; *CACNA1AH, TRPM4[B], NOTCH1, LTBP2, DSP, LAMA2, APO4*, and *ANK2* in gene panel) that carried many variants, but only a small fraction were annotated as likely pathogenic. Conversely, genes that did not tolerate mutations had a similar number of total and likely pathogenic variants. The list of genes displayed in Figures 2C,D only overlaps partially because many of the exome-only loci carried more variants than gene panel ones (note the different scale in *y*-axis).

### Do Likely Pathogenic Mutations Cooccur?

**Figure 3:**
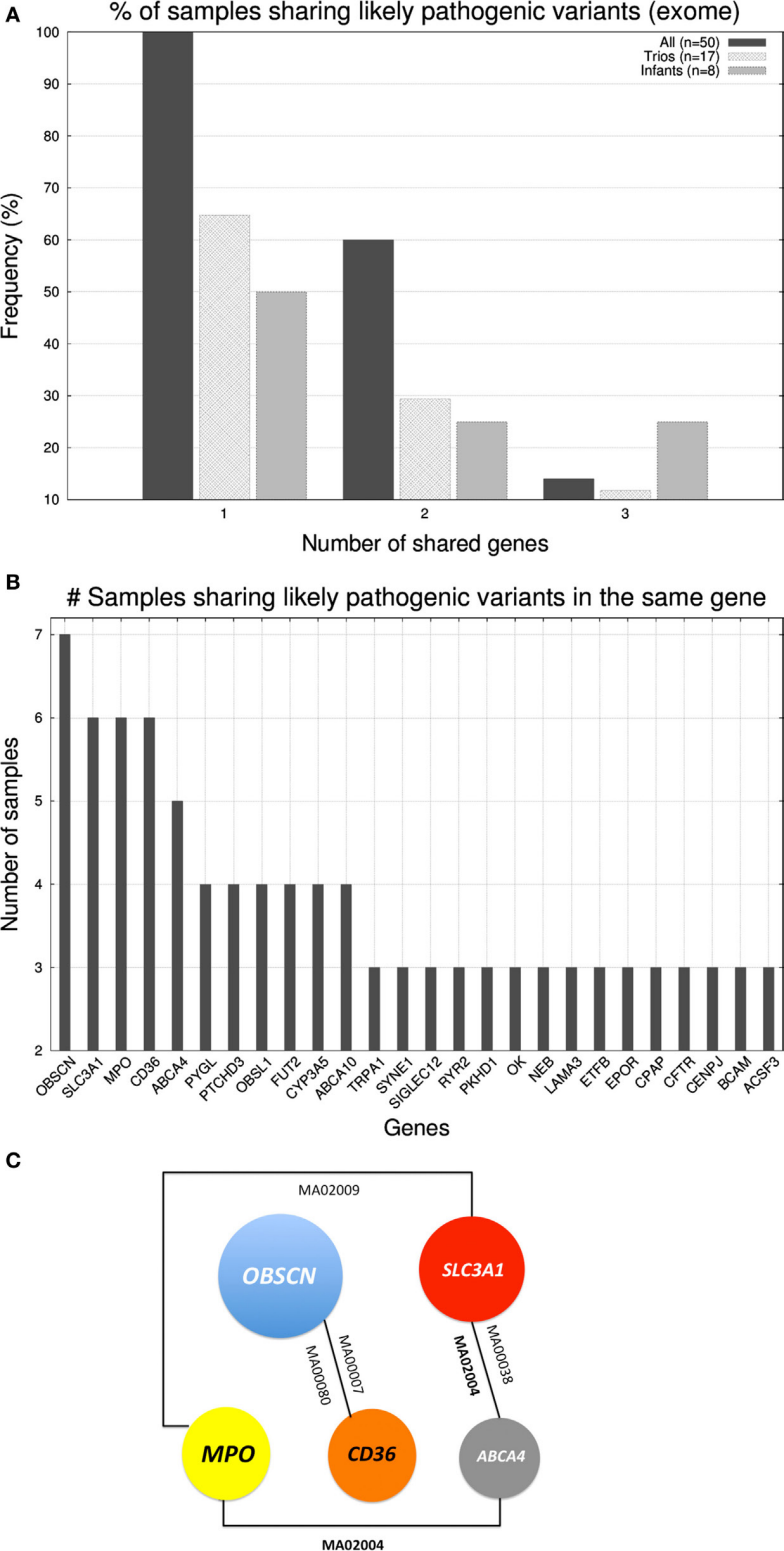
**(A)** Histogram displaying the percentage of samples that shared up to three genes (with likely pathogenic variants) with at least another sample. **(B)** Number of samples sharing genes with likely pathogenic variants (exome level). **(C)** Schematic representation of the cooccurrence of the five most shared genes (shared by ≥5 samples).

After studying the distribution of variants in genes we investigated whether there existed cooccurrence of pathogenic variants in the same genes across individuals. In the case of gene panel, only a few cases carried >1 likely pathogenic variants being the affected genes dissimilar across individuals. In case of the exome, in Figure 3A, we represented the percentage of samples that shared up to three genes (with likely pathogenic variants) with at least another sample. Only 7 out of 50 samples shared three genes, 3 shared two, and all 50 samples shared at least one gene with another sample. Trios and infants had lower percentages due to the smaller group size, which limited the probability of two samples carrying mutations on the same genes. With regard to which were the genes were shared, in Figure 3B, we show the distribution of genes according to the number of samples that carried likely pathogenic mutations in them. The five most abundantly mutated loci were: *OBSCN* (obscurin, cytoskeletal calmodulin, and titin-interacting RhoGEF) that plays a role in the organization of myofibrils during sarcomere assembly and has been associated with HCM, *SLC3A1* (neutral and basic amino acid transport protein rBAT) that is associated with cystinuria, *MPO* (myeloperoxidase) that has been associated with coronary artery disease, *CD36* (cluster of differentiation 36) a class B scavenger receptor implicated in cardiovascular disease, and *ABCA4* [ATP-binding cassette, subfamily A (ABC1), member 4] that has been associated with macular degeneration (see Figure 3C). None of the five protein products of these genes are part of the same biological network.

### Is There a Genetic Fingerprint for Sudden Death?

The cardiovascular forensic examinations of each case often reveal complex phenotypes. For instance, a given individual could have a mixture of HCM, dilated cardiomyopathy, and the fibrofatty replacement typical from arrhythmogenic right ventricular cardiomyopathy/dysplasia (ARVC/D). In terms of genetics variants, it is not uncommon to identify variants in genes associated with malignant arrhythmias along with mutations in genes associated with heart muscle disease (25). Under the assumption that similar genotypes will yield similar phenotypes, we investigated if the combination of affected genes could be used to classify samples.

**Figure 4:**
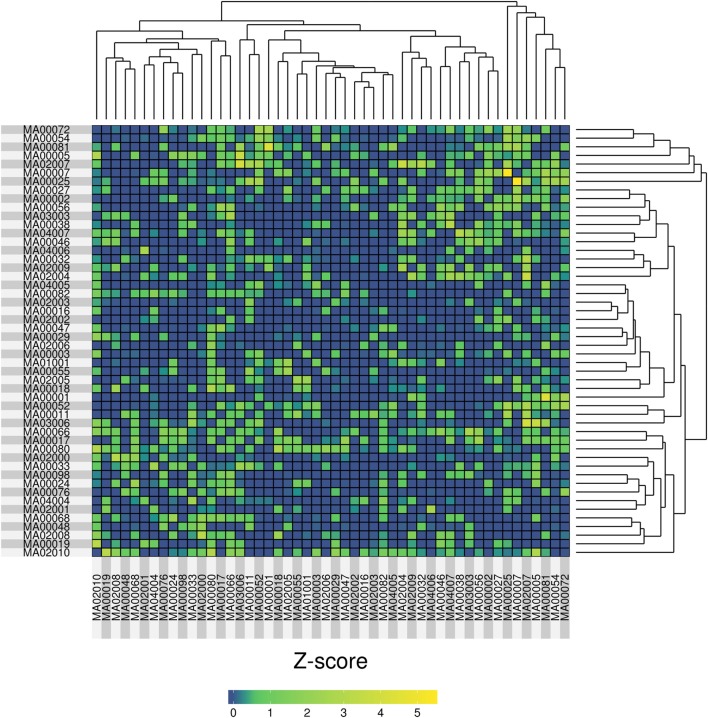
Heatmap representation of the pairwise similarity (measured by a Jaccard index, standardized with a *Z*-score) among 50 molecular autopsy cases. The Jaccard index was computed using exome genes that carried variants in SG-ADVISER categories 1–3.

In order to compare the samples we used a genetic fingerprint, created as follows: for each sample, we collapsed all variants (per gene) that fell into SG-ADVISER categories 1–3. We included SG-ADVISER category number 3 (variant is previously unreported and is of the type which may or may not be causative of the disorder) to increase the number of variants, as categories 1–2 yielded only a maximum overlap of three genes between two individuals (see above). Then, we used that gene list to carry out a pairwise (sample-based) comparison using an intersection over union index (Jaccard index), standardized with a *Z*-score. The resulting matrix of pairwise *Z*-scores was transformed to a heatmap with R software (44) on which we applied hierarchical clustering (Figure 4). After standardization, we obtained eight pairwise comparisons having *Z*-scores >3 resulting from the intersection of 6–10 genes over the union of 88–116. The highest *Z*-score = 5.44 was obtained between cases MA00007 and MA00025 (both forensic autopsy negatives) with an intersection of 10 genes (*ASPM, CCDC88C, IDUA, KCNK18, MAST4, MYO15A, PKHD1, SPTBN2, SPTBN5*, and *SYNE1*) over a union of 96.

### Mitochondrial DNA (mtDNA) Variants

Due to the fact that cardiomyocytes are in constant need for energy, all the steps involved in ATP generation are of crucial importance. It is well established that mitochondria play roles in many cardiomyopathies (45), and it has been suggested that depletion of ATP (caused by an external stressor) in defective mitochondria might play a role in some forms of sudden infant death syndrome (10, 46). Despite that, downstream analysis of mtDNA variants is rarely performed in exome-based data (47). Here, we used our bioinformatics expertise in analysis of mtDNA data [methods described elsewhere (47)] to add this information to each case. After the variant calling, we only considered mtDNA variants that possessed a heteroplasmic fraction (HF) >0.2 and a MAF <0.05 in 1,000 Genomes (36).

**Figure 5:**
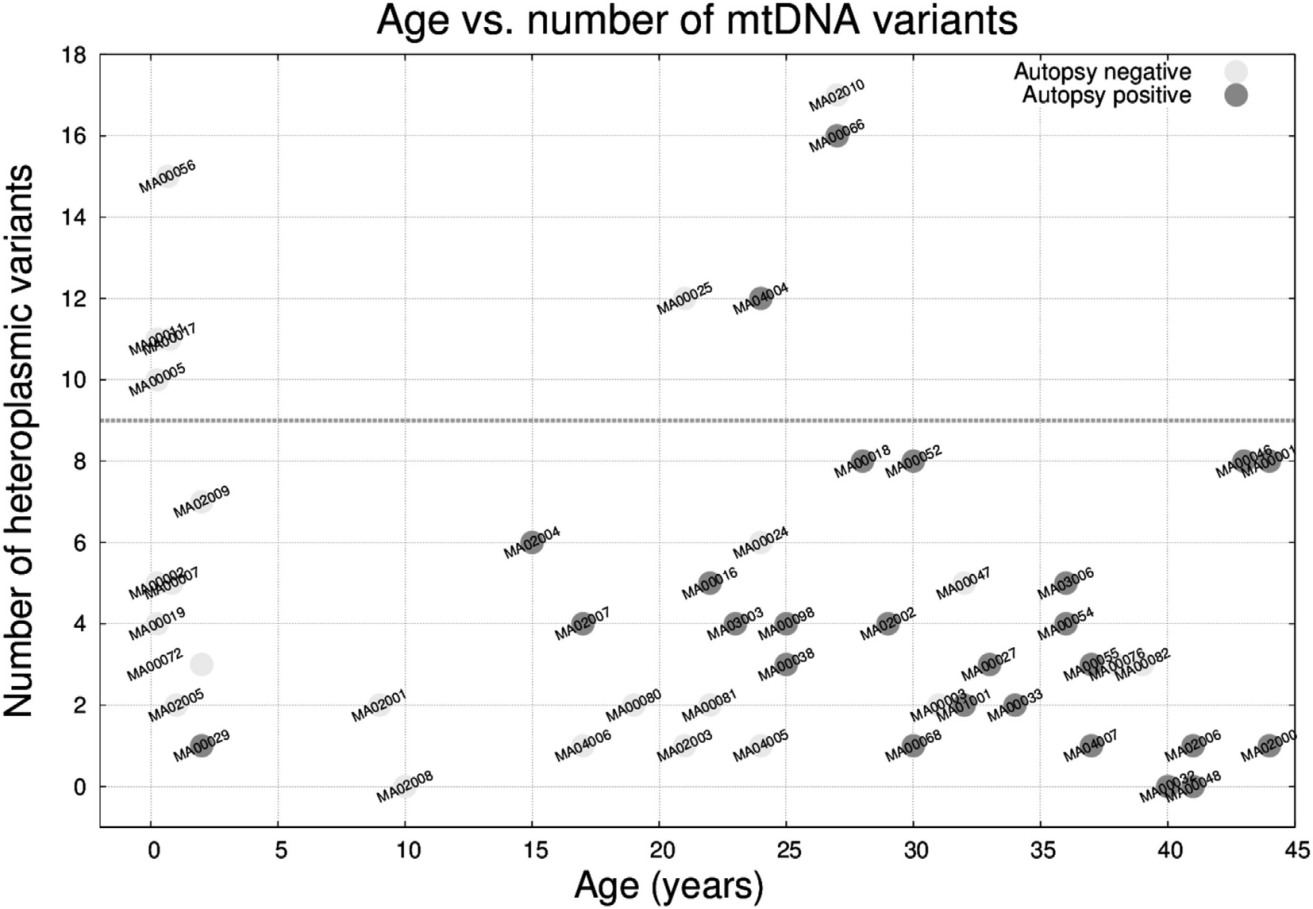
Age versus number of mitochondrial DNA heteroplasmic variants in the 50 molecular autopsy cases colored according to being forensic autopsy positive or negative. Note that 4 out 8 infants plus 4 other cases carried ≥10 variants.

The median number of heteroplasmic variants per case was 4 (interquartile range: 2–7, min value: 0, max value: 17). It is worth mentioning that the number of variants was not associated with differences in depth of coverage relative to the sequencing step (Figure S1 in Supplementary Material). We compared the number of heteroplasmic variants per sample with respect to age (Figure 5) and we observed that there was no relation between the two parameters, and that 8 (out of the 50) individuals carried ≥10 heteroplasmic variants. From those eight cases, four were infants (half of the infant group). A high percentage of the variants were in the *MT-DLOOP* region (Table 3), a locus recently associated with total levels of heteroplasmy (47). The heteroplasmic variants were not *de novo*, as they were also present in the mothers with equally high levels of HFs.

**Table 3:** Values for the eight molecular autopsy cases who carried ≥10 mtDNA heteroplasmic variants.

ID (age/sex/race)	Proband	Mother	Father	Forensic autopsy	Nuclear variants (ClinVar)	mtDNA variants (ClinVar)

	All/MT-DLOOP	All (shared)/*MT-DLOOP* (shared)	All (shared)/*MT-DLOOP* (shared)			
MA02010 (27Y/Male/African-American)	17/5			–	*TTR* (amyloidosis)	*MT-ND1* (SIDS)^a^
MA00066 (27Y/Female/African-American)	16/5			Aneurysm	–	*MT-ND1* (SIDS)^a^
MA00056 (8M/Female/Hispanic)	15/9	15 (15)/9 (9)	6 (6)/4 (4)	–	–	*MT-TC, MT-TT*
MA04004 (24Y/Male/White)	12/4			HCM	–	*MT-RNR1* (HCM)
MA00025 (21Y/Male/NA)	12/7			–	–	*MT-TQ*
MA00017 (9M/Male/African-American)	11/4	NA	NA	–	*HCN4* (BrS)	*MT-TA, MT-TL2*
MA00011 (3M/Male/White)	11/5	11 (11)/5 (5)	0 (0)/0 (0)	–	*RYR2* (CPVT)	*MT-ND5* × 3 (LHON)
MA00005 (3M/Male/White)	10/4	10 (10)/4 (4)	0 (0)/0 (0)	–	–	*MT-TE, MT-ND5* × 2 (LHON)

*BrS, Brugada syndrome; CPVT, catecholaminergic polymorphic ventricular tachycardia; HCM, hypertrophic cardiomyopathy; LHON, Leber hereditary optic neuropathy; SIDS, sudden infant death syndrome*.

*^a^Verified by Sanger sequencing*.

## Discussion

Here, we analyzed 50 molecular autopsies of sudden unexpected and unexplained cases of cardiac death in the young (<45 years old) predominantly from the San Diego County. In keeping with previous studies of SDY, we observed a 1:2 female to men ratio (5, 22, 25, 27). The number of cases had a peak in infants, and exponentially increased up to the ages studied (2, 25). A surprisingly large percentage of sudden infant death syndromes happened during the middle of the week (Wednesdays and Thursdays), whereas for older individuals, there was a peak on Mondays, a day known for being enriched in sudden deaths due to ischemic events (1). Friday was the day less prone to deaths. While a relation between SCD and variation in weekly stress levels can be speculated in young adults with social life, such a connection seems less intuitive for infants. In that regard, it is known that heart rate and ventilation tends to synchronize in individuals sharing space (48–50), and it is also known that maternal environment (e.g., cortisol in breast milk) can influence infant development (51, 52). Given the limited sample size, we could not establish a legitimate explanation for the excess of infant deaths during the middle of the week. However, we cannot discount the possible contribution of environmental factors. We found that for the entire cohort (*n* = 50), the majority of deaths occurred between 8 a.m. and 8 p.m., a window that overlaps with previous findings [6 a.m. to 12 p.m. and late afternoon (1, 53)]. This circadian relationship has been associated with sympathetic activation (54) and protein regeneration (55). The deaths were not equally spread during the year, as we found a peak in April, followed by months of June, October, and September. Only two cases of SDY happened in January and May. With the exception of the lower mortality found in late summer, our seasonal findings do not overlap with previous studies on SCD (56), but it is important to remember that our cohort consisted of young individuals and did not include deaths by ischemic events *per se*. Molecular autopsies overall gave a modest yield in terms of likely pathogenic variants, irrespective of being performed in forensic autopsy positive or negative cases. A high percentage of the variants found fell under the category of plausible or VUS (15, 57). Still, combined use of forensic and molecular autopsies provided the maximum diagnostic yield.

We compared an exome-based approach to a simulated gene panel for cardiac disease. In that regard, we found that exome-based analysis led to many likely pathogenic variants that could potentially explain sudden death. However, due to the lack of robust databases for variants associated with SDY, we could not make conclusive statements since the genes themselves have not been associated with sudden death and we only have one off cases in these instances. On the other end of the spectrum, gene panel-based analysis provided a manageable number of likely pathogenic mutations, but the overall diagnostic yield was low. An obvious advantage of using an exome-based capture is that it allows for searches using lists of genes and opens the possibility of expanding the lists without repeating the sequencing experiments, but at the expense of losing depth of coverage (37, 58–61). The effect of other variants not studied here, such as somatic mutations, variants in non-coding regions, and copy number variations, is currently an active field of cardiovascular research (6, 62–65).

In terms of where the variants fall in the nuclear exome, the tendency was that larger genes (e.g., *TTN*) accumulated more variants. Regardless of size, some genes are less tolerant to mutations and this information can be useful to assess pathogenicity likelihood when an unseen variant is found (41–43). Even for recognized Mendelian diseases such as long QT syndrome, Brugada syndrome, or catecholaminergic polymorphic ventricular tachycardia, we often observed that multiple variants could potentially be contributing to the observed phenotype. In that sense, the separation of VUS with respect to true pathogenic variants is probably the most challenging task that the field of genetics will face during the upcoming years. In terms of sample classification, the small sample size and the lack of consistency on genes carrying pathogenic variants caused that a comparison based on a genetic fingerprint did not perform satisfactorily. Still, and under the assumption that similar genotypes should lead to similar phenotypes (10, 66), we believe that in the future, when larger cohorts with clinical ontologies become accessible (67), this departure from a “n-of-1” might help us see the forest through the trees and will facilitate the diagnostic of disease. In the case of cardiomyopathy, the cohort does not necessarily need to be restricted to deceased individuals (25), as it is possible to perform early screening in at risk populations (68).

Finally, we analyzed mtDNA variants from all 50 samples (and trios when available) and observed that 50% of the infants (4 out of 8) had a substantial increase in the number of heteroplasmic variants (≥10 variants) with respect to the rest of the cohort (median: 4; interquartile range: 2–7). The variants were not *de novo* (mothers were also carriers yet apparently asymptomatic) and had high heteroplasmic levels. Among those variants, only a subset (as annotated by external databases) was capable of producing mitochondrial disease. In that sense, we contemplate the possibility that an excess of heteroplasmic mtDNA variants might confer susceptibility for a mitochondrial “system-failure” under conditions of metabolic stress, especially when an immature metabolism is at play. Parenthetically, we recently found that people over 100 years free from disease tend to have low number of heteroplasmic variants (47), which supports the idea that heteroplasmic levels might be associated with metabolic efficiency. This is just a hypothesis since we cannot assess to what extent mtDNA variants contributed to the sudden death. We acknowledge that our cohort is small and that our findings need to be replicated. If heteroplasmy turns out to be associated with metabolic disadvantage, implementing a preconceptual/prenatal genetic test for risk for sudden infant death syndrome based on mtDNA should be straightforward.

We believe a comprehensive effort to collect and share genetic and phenotypic data is needed in order to define pathogenic variants more precisely, provide quantifiable risks to living relatives, and unravel the incomplete penetrance, variable expressivity, and gene–environment interactions evident in previous findings. The aggregate genomic data for all cases can be accessed at https://genomics.scripps.edu/browser.

## Conclusion

We present here a systematic analysis of the first 50 cases from our MA for sudden death in the young study. We found that males were affected twice as often as females, and that deaths followed circadian, weekly and seasonal patterns. Molecular autopsies identified a likely causal variant in 14 cases, yielding maximum value when combined with forensic ones. Almost all the reported likely causal variants were in genes associated with cardiac disease, thus, “escalating” to exome did not improve the diagnostic yield. In that regard, most of the unreported variants were VUS. At the coding level, our cohort did not have many individuals sharing genes with pathogenic variants and thus a genotype-based classification was unsatisfactory. Adding mtDNA variants to molecular autopsies provided new insights as well as new uncertainties. We found that half of the infants carried out an unusually high number in heteroplasmic variants. Overall, the analysis of MA cases at the cohort-level adds a new dimension to the understanding of the genetic causes of SDY. We believe that a global effort to share genomic data in a centralized knowledge resource is needed to succeed in transitioning MA negatives to positives.

## Ethics Statement

This study was carried out in accordance with the recommendations of Scripps Office for the Protection of Research Subjects, Protocol number IRB-14-6386 written or verbal consent was obtained from each subject or their authorized representative.

## Author Contributions

MR designed, analyzed, interpreted the data, and wrote the manuscript. JW and ST participated as clinical trials coordinators. TP performed laboratory experiments. EM provided case review and revised the manuscript. JL and GW conceived the study, oversaw forensic autopsies, and provided case review. ET conceived the study, obtained funding, and organized the collaboration with the medical examiner. AT obtained funding, interpreted the data and revised the manuscript.

## Conflict of Interest Statement

The authors declare that the research was conducted in the absence of any commercial or financial relationships that could be construed as a potential conflict of interest.
