# Corrigendum: Molecular Autopsy for Sudden Death in the Young: Is Data Aggregation the Key?

**DOI:** 10.3389/fcvm.2017.00090

**Published:** 2018-01-24

**Authors:** Manuel Rueda, Jennifer L. Wagner, Tierney C. Phillips, Sarah E. Topol, Evan D. Muse, Jonathan R. Lucas, Glenn N. Wagner, Eric J. Topol, Ali Torkamani

**Affiliations:** ^1^The Scripps Translational Science Institute, Scripps Health, The Scripps Research Institute, La Jolla, CA, United States; ^2^Division of Cardiology, Scripps Clinic, La Jolla, CA, United States; ^3^Medical Examiner Department, San Diego County, San Diego, CA, United States

**Keywords:** molecular autopsy, sudden cardiac death, whole exome sequencing, gene panel, mitochondrial DNA

In the original article, the online version of Table S1 in Supplementary Material was not the final one. The online version of Table S1 in Supplementary Material has been updated along with the present corrigendum.

The authors apologize for this oversight. The updated information does not change the scientific conclusions of the article.

The original article was updated.

## Conflict of Interest Statement

The authors declare that the research was conducted in the absence of any commercial or financial relationships that could be construed as a potential conflict of interest.

