# Supplementary material for: Molecular Autopsy for Sudden Death in the Young: Is Data Aggregation the Key?
Source: Front Cardiovasc Med. 2017 Nov 9;4:72. doi: 10.3389/fcvm.2017.00072 (PMC5694161; doi:10.3389/fcvm.2017.00072)
Supplement: Supplementary file 4 [file Image_1.PDF]

SUPPORTING FIGURE S1

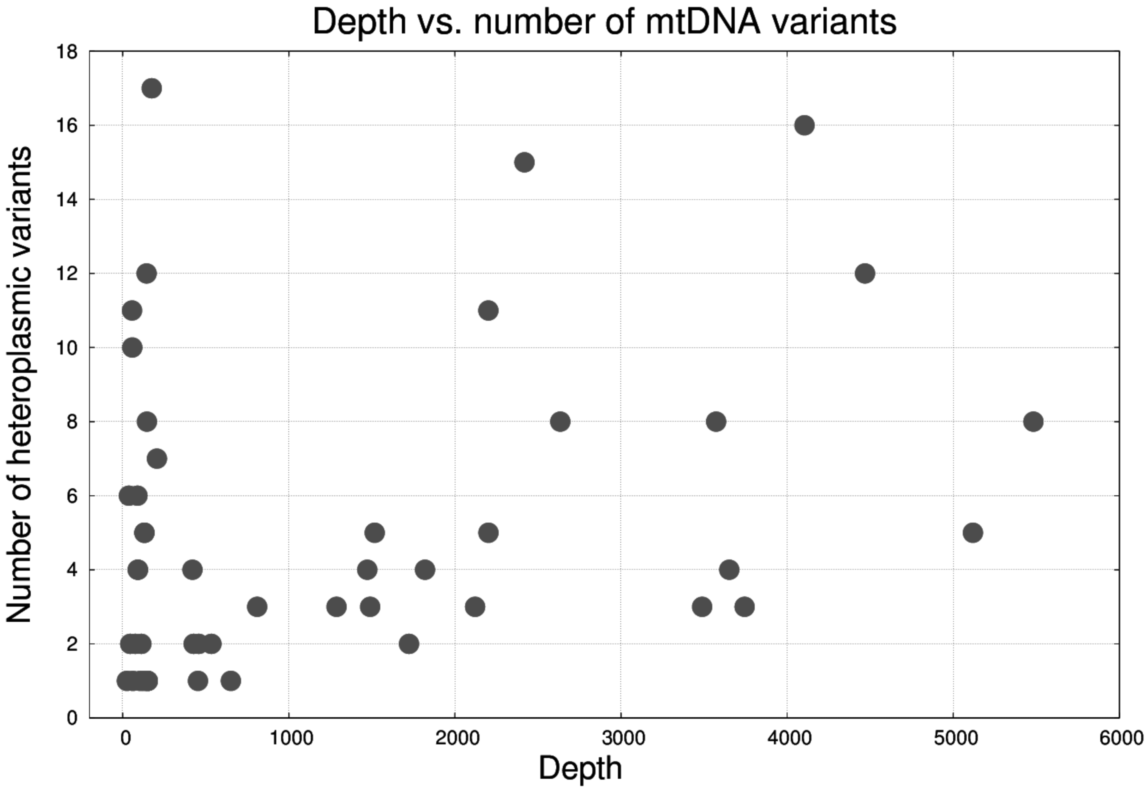

Supporting Figure S1. Scatter plot of the average (per position) sample depth vs. the number of heteroplasmic variants for the 50 Molecular Autopsy cases.
